# Supplementary material for: Orbital Adipose Tissue: The Optimal Control for Back-Table Fluorescence Imaging of Orbital Tumors
Source: Bioengineering (Basel). 2024 Sep 14;11(9):922. doi: 10.3390/bioengineering11090922 (PMC11428325; doi:10.3390/bioengineering11090922)
Supplement: Supplementary file 1 [file bioengineering-11-00922-s001.zip › bioengineering-3119229-supplementary.pdf]

Orbital Adipose Tissue: The Optimal Control for Back-Table Fluorescence Imaging of Orbital Tumors

Lan Yao[#1], Wenhua Zhang[#2], Xuedong Wang[5#], Lishuang Guo[3,4], Wenlu Liu[1], Yueyue Li[1], Rui Ma[1], Yan Hei[1], Xinji Yang*[1], Zeyu Zhang*[3,4], Wei Wu*[1]

Supplementary Table S1. Characteristic of all the patients.

Patient Number	Gender	Age（Years）	Weight（kg）	Pathology	Control Tissue
1	female	58	49	pleomorphic adenoma of the lacrimal gland	adipose
2	female	35	50	non-Hodgkin’s lymphoma	adipose
3	female	67	73	malignant melanoma	adipose
4	female	66	65	malignant melanoma	adipose
5	male	69	70	lymphoproliferative disorder	adipose
6	male	67	70	non-Hodgkin’s lymphoma	adipose
7	male	77	92	pleomorphic adenoma of the lacrimal gland	adipose, muscle
8	male	47	69	neurofibroma	adipose
9	male	47	74	schwannoma	adipose
10	male	31	75	pleomorphic adenoma of the lacrimal gland	adipose
11	male	27	84	neurofibroma	adipose
12	female	52	65	lymphoproliferative lesions with atypical lymphocytosis	adipose
13	male	48	78	pleomorphic adenoma of the lacrimal gland	adipose
14	female	46	60	schwannoma	adipose
15	male	52	84	spindle cell tumor	muscle
16	male	48	76	optic nerve glioma	adipose
17	female	64	69	reactive lymphoid hyperplasia	adipose
18	male	30	47	neurofibroma	muscle
19	male	18	66	pleomorphic adenoma of the lacrimal gland	adipose
20	male	19	49	neurofibroma	adipose
21	female	77	51	seborrheic keratosis	skin
22	female	37	65	liposarcoma	adipose
23	female	36	60	B-cell lymphoma	adipose
24	male	64	90	squamous cell carcinoma	adipose
25	male	20	57	pleomorphic adenoma of the lacrimal gland	adipose
26	female	41	59	lymphangiomatous lymphoma	adipose
27	male	54	72	moderately differentiated adenocarcinoma	adipose
28	female	74	55	schwannoma	adipose
29	female	56	49	malignant tumor of the lymphatic and hematopoietic system	adipose
30	female	80	52	dermatofibrosarcoma protuberans	adipose
31	male	54	80	inflammatory lesion	adipose
32	male	33	93	pleomorphic adenoma of the lacrimal gland	adipose
33	female	55	55	liposarcoma	adipose

34	female	72	65	schwannoma	adipose, periosteum
35	male	60	70	myoepithelial carcinoma	adipose, periosteum
36	female	66	80	intramuscular hemangioma	adipose
37	female	64	70	lymphangiomatosis	adipose, periosteum
38	male	80	66	skin basal cell carcinoma	skin
39	female	69	66	sebaceous gland carcinoma	skin
